# Evaluation and correlation of heart rate variability and ventricular repolarization parameters in an Indian pediatric clinical hypothyroid population: a prospective cohort study

**DOI:** 10.1038/s41598-026-36745-2

**Published:** 2026-01-29

**Authors:** Divyam Dhakar, Himani Ahluwalia, K. R. Meena, Akash Tomar

**Affiliations:** 1 Department of Physiology , Vardhman Mahavir Medical College and Safdarjung Hospital , New Delhi, India; 2 Department of Pediatrics , Vardhman Mahavir Medical College and Safdarjung Hospital , New Delhi, India; 3 Department of Physiology, Kasturba Medical College Manipal, Manipal Academy of Higher Education , Manipal, India

**Keywords:** Heart rate variability, Ventricular repolarization, Levothyroxine therapy, Pediatric hypothyroidism, Child health, Cardiology, Endocrinology, Medical research, Physiology

## Abstract

To compare ventricular repolarization and heart rate variability in hypothyroid and healthy children and to assess the effects of levothyroxine therapy on cardiac electrophysiological and autonomic parameters. This study investigated ventricular repolarization parameters and heart rate variability (HRV) in hypothyroid children (5–12 years) compared with age-matched healthy controls and patients before and after thyroxine treatment. This study included 64 participants divided into hypothyroid and healthy groups (32 each). Parameters such as the Tpe interval, QT interval, and HRV indices were measured via standard electrocardiographic and HRV analysis techniques. Thyroid hormone levels were measured. Post treatment measurements were also performed on hypothyroid children following 3 months of levothyroxine therapy. Hypothyroid children presented significantly prolonged Tpe intervals (58.07 ± 9.21 ms vs. 53.80 ± 8.89 ms, p = 0.039), reflecting increased heterogeneity in ventricular repolarization. HRV analysis revealed decreased parasympathetic modulation (e.g., lower RMSSD and pNN50 values) in hypothyroid participants. Posttreatment improvements in HRV indices and thyroid hormone levels correlated with normalization of cardiac electrophysiological parameters. This study highlights that even mild thyroid dysfunction in children can subtly disrupt cardiac electrical stability and autonomic balance which may increase the risk of arrhythmia in this population. Timely thyroxine therapy not only restores thyroid levels but also powerfully rebalances the electrical and autonomic balance of the heart. These findings reaffirm the critical need for early detection and proactive treatment of pediatric hypothyroidism to safeguard lifelong cardiovascular health.

**Supplementary Information:**

The online version contains supplementary material available at 10.1038/s41598-026-36745-2.

## Introduction

Overt or clinical primary hypothyroidism is defined as thyroid-stimulating hormone (TSH) concentrations above the reference range and free thyroxine concentrations below the reference range^1^. In the NHANES III study, the overall prevalence of hypothyroidism was 4.6%. The overall disease burden of hypothyroidism in sub-Saharan Africa, on the basis of largely hospital clinic data, is predicted to be minimal (or even rare) and substantially lower than the prevalence reported in African Americans. Regional variations have been reported in India, with higher rates of hypothyroidism in inland regions than in coastal regions^2^.

Hypothyroidism is associated with multisystem involvement, but its cardiovascular effects are particularly important. Thyroid hormone deficiency leads to bradycardia, reduced myocardial contractility, diastolic dysfunction, and altered vascular resistance, often contributing to systemic hypertension and dyslipidemia^3^. These changes increase cardiovascular morbidity and predispose to arrhythmia. In addition, hypothyroidism has been linked to disturbances in autonomic regulation, reflected by altered heart rate variability (HRV), and to abnormalities in ventricular repolarization parameters, which may increase the risk of sudden cardiac events. A table showing the different effects of hypothyroidism on the human body is shown in Supplementary Text 1 (S1).

Thyroid hormones and TSH influence cardiac contraction, myocardial oxygen consumption, and cardiac output, thereby playing a vital role in maintaining cardiovascular homeostasis^4^. Heart rate variability (HRV) is a widely used noninvasive tool for evaluating autonomic regulation of the heart^5^. In this regard, enhanced parasympathetic activity is associated with reduced susceptibility to malignant arrhythmias, while increased sympathetic drive has been linked to fatal ventricular arrhythmias^6^. Ventricular repolarization can also be assessed using the interval from the peak to the end of the T wave (Tp-e), with Tp-e/QT and Tp-e/QTc ratios recognized as markers of ventricular arrhythmogenesis, as suggested by earlier studies on large cohorts^7,8^ Together, HRV and Tp-e/QT indices provide valuable insights into autonomic balance and repolarization heterogeneity, serving as predictors of arrhythmic risk in hypothyroid states.

Cardiac electrophysiology is known to be affected in both children and adults with clinical hypothyroidism^9^, although studies in the literature on cardiac electrophysiology in children with hypothyroidism are limited in number, and studies in adults have reported contradictory results^10,11^. Thus, this study aims to investigate the interplay between altered autonomic functions and cardiac ventricular repolarization parameters (mainly Tp-e interval and ratios) in the pediatric population of individuals with clinical hypothyroidism. These findings could also provide insight into how thyroid hormones impact cardiovascular parameters in a hypothyroid child’s life.

## Methods

### Study group

Thirty-two patients (20 boys and 12 girls) aged between 5 and 12 years who were followed up with a diagnosis of clinical hypothyroidism in the Department of Pediatrics, Vardhman Mahavir Medical College and Safdarjung Hospital, New Delhi, were included in the study. The control group consisted of 32 healthy children and adolescents (25 boys and 7 girls) who came to pediatric outpatient clinics with nonspecific symptoms and who were similar to the study group with respect to age distribution. The study was performed prospectively. Power analysis was used to determine the statistical robustness of the study.

### Study protocol

All the subjects were called to the Autonomic Function Laboratory, Department of Physiology, in the morning hours, and all the investigations were carried out between 9 a.m. and 11:30 a.m. All the parents/guardians were informed of the prerequisites for HRV, and informed consent was obtained. The subjects were instructed to refrain from caffeine/tea ingestion on the day of the investigations and to come after having a light breakfast 2 h prior to testing. The temperature of the lab was maintained between 23 °C and 25 °C. All the subjects were tested under similar laboratory conditions^12^. They were allowed to adapt to experimental and environmental conditions for 20 min. The nature of the tests was explained to the subjects beforehand, and informed consent was obtained.

### Ethics declarations

The parents of the children included in the study and the children were informed about the purpose and method of the study, and the human ethics and consent to participate were obtained regarding their voluntary participation. The study was approved by the Institutional Ethics Committee of VMMC & Safdarjung Hospital (IEC/VMMC/SJH/Thesis/2020-11/CC-262) and dated 10/12/2020. The procedures adhered to the guidelines outlined in the Declaration of Helsinki 2013, and this study was conducted and reported in accordance with the Strengthening the Reporting of Observational Studies in Epidemiology (STROBE) guidelines for cohort studies.

### Parameters measured

#### Anthropometric and laboratory parameters

The anthropometric measurements recorded were height in cm (by stadiometer attached in the laboratory), weight in kg (by weighing machine) and body mass index (BMI) = weight (in kg)/(height in meters)^2^. All values were measured in subjects with comfortable clothing and who were barefooted.

The institutional laboratory criteria for hypothyroidism - the range of normal values of the level of thyroid hormones in serum as per those followed in the Department of Laboratory Medicine, VMMC & SJH are T3 0.69–2.02 ng/ml, T4 4.4–11.6 µg%, and TSH 0.4–6.2 mIU/ml. Thyroid hormone levels were recorded from the case sheets of hypothyroid patients. A 5 ml blood sample was taken from controls for estimation of thyroid hormone levels, and after 8 h of fasting, it was obtained through outpatient clinic visits. The immunochemiluminometric method with Elecsys kits (Roche, Mannheim, Germany) was used to measure these values.

#### Heart rate variability

HRV recordings were obtained via an ML 870B80 (A.D. Instruments) (AD Instruments, Australia). Time-domain and frequency-domain analyses were performed for all patients and controls. The analysis was done using the II lead. The HRV was calculated from 5-minute ECG recordings according to standard practices automatically by the Power Lab software incorporated in ML 870B80^13^. The standard deviation of all RR intervals (SDRR), standard deviation of the averages of NN intervals in 5-minute segments of the recording (SDARR), square root of the mean of the sum of the squares of differences between adjacent NN intervals (RMSSD), mean of the standard deviations of all NN intervals for all segments of the entire recording (SDNN index), number of pairs of adjacent RR intervals differing by more than 50 ms in the entire recording (RR50) and RR50 count divided by the total number of all RR intervals (pRR50) were calculated in time domain analyses. Frequency domain parameters were obtained in the first 5-minute recordings of the patient and control groups. Low-frequency (LF), high-frequency (HF), and LF/HF ratios with normalized units were calculated.

#### Electrocardiogram

Twelve-lead ECGs were obtained from all participants in the hypothyroid and control groups via an ML 870B80 (A.D. Instruments) (AD Instruments, Australia). The longest and shortest QT intervals, longest and shortest P wave durations, Tp-e duration, heart rate, and PR durations were measured from lead V5 by the software associated with ML 870B80^14^. QT intervals were measured in lead V5 from the beginning of the QRS complex to the end of the T wave. When a U wave is present, if large (i.e., > 50% amplitude of the T wave) and merging into the T wave, it should probably be included, but excluded if it is smaller or separate^15^. When the end of the T wave could not be determined, the lead was not included. Additionally, QT interval measurements were corrected via the Bazett formula (QT/√RR) and recorded. The Tpe/QT and Tpe/QTc ratios were calculated via these measurements. Bazett formula was used as it is the most common correction method used in populations of this region^16^. All statistical analyses were performed by a statistician who was blinded to group allocation to minimize analytical bias.

### Inclusion criteria

- Newly diagnosed cases of hypothyroidism in children aged 5–12 years, as well as normal children within the same age group.

### Exclusion criteria

- Children with a history of congenital hypothyroidism or other congenital abnormalities.
- Those on medications affecting thyroid function or ECG parameters (e.g., antimalarials, antipsychotics, ketoconazole, or antiarrhythmics); and children with chronic conditions such as kidney, liver, endocrine, cardiovascular, or respiratory diseases; severe anemia.
- Children suffering from acute illnesses or taking medications for common ailments such as cough, cold, or fever were excluded.
- Children who have undergone radiation therapy or thyroidectomy, as well as those who are morbidly obese, malnourished, or have any skeletal deformities, particularly of the chest.
- Any other chronic medical or surgical conditions.

### Statistical analysis

GraphPad Prism 10.0 was used for data analysis. Data on quantitative (numerical) variables that had a normal distribution are shown as the mean ± SD, whereas those that did not have a normal distribution are shown as the median (Q1-Q3). Categorical variables are presented as frequencies and percentages (%). The normality of the data was tested via the Kolmogorov‒Smirnov test. Parametric and nonparametric tests were applied accordingly. Unpaired t tests or Mann‒Whitney tests were performed to compare quantitative variables between the two groups. A paired t test was performed to compare quantitative variables between hypothyroid children at the time of diagnosis and those after 3 months of thyroid replacement treatment. The Pearson correlation coefficient/Spearman rank correlation coefficient was used to determine the correlation between the thyroid profile and ECG values via power lab software and HRV parameters. The level of significance was considered *p* < 0.05, the confidence interval was 95%, β = 0.2, and the power was considered 80%. A post-hoc power analysis was also performed for the follow-up comparisons (paired data, *n* = 23) using the observed effect sizes; the achieved power for significant parameters such as the Tpe interval, Tpe/QT ratio, and Tpe/QTc ratio was above 80% at α = 0.05.

## Results

For this study, 64 subjects, including both males and females aged 5–12 years, were assessed with due consideration to the inclusion and exclusion criteria. Recordings were performed for all 32 subjects in each group of hypothyroid (at the time of diagnosis) and healthy children. All the above parameters were also recorded in the hypothyroid children after three months of thyroid replacement therapy (Table 1).

### Baseline characteristics of the subjects

Anthropometric measurements such as age, height, weight and body mass index were recorded for both groups. There was no statistically significant difference in age or anthropometric parameters between the two groups, as shown in Table 1. The hypothyroid group included 20 males and 12 females. In the normal subject group, there were 25 males and 7 females, and the percentages of males and females were 62.5% and 37.5%, respectively, among the hypothyroid children and 78.12% and 21.87%, respectively, among the normal children.

**Table 1:** Baseline characteristics of the study population.

Variable	Hypothyroid (Mean *±* SD)	Normal (Mean *±* SD)	Difference in Means (Δ)	*p* value
Age in years	8.88 ± 1.88	9.31 ± 1.96	−0.43	0.320
Weight in Kgs	40.78 ± 7.89	42.25 ± 6.80	−1.47	0.443
Height in cm	131.06 ± 12.05	133.66 ± 9.70	−2.60	0.386
BMI in kg/m^2^	23.62 ± 2.59	23.58 ± 2.54	+ 0.04	0.946
T3 (ng/ml)	0.45 ± 0.30	1.23 ± 0.46	−0.78	0.001**
T4 (µg%)	2.51 ± 0.92	8.20 ± 1.82	−5.69	0.001**
TSH (mIU/ml)	120.89 ± 53.82	3.20 ± 1.87	+ 117.69	0.001**
Resting Heart Rate	80.61 ± 12.15	77.40 ± 11.83	+ 3.21	0.600
SBP in mm Hg	109.88 ± 6.25	110.00 ± 5.39	−0.12	0.995
DBP in mm Hg	77.38 ± 4.61	77.47 ± 4.14	−0.09	0.893

p value* significant at < 0.05. *BMI* body mass index, *TSH* thyroid stimulating hormone, *SBP* systolic blood pressure, *DBP* diastolic blood pressure.

### Electrocardiographic and HRV parameters of the study subjects

ECG parameters such as the Tpe interval, QT interval, QTc interval, Tpe/QT ratio, Tpe/QTc ratio, PR interval and JT interval were recorded in both groups. The time domain parameters measured in our two study groups were the average RR, median RR, SDRR, SDSD, RMSSD and PRR50. The frequency domain parameters measured in our study were total power, VLF power, LF power, HF power, VLF power%, LF power%, HF power%, normalized LF power (LFnu), normalized HF power (HFnu) and the LF/HF ratio. None of the HRV parameters were significantly different between the two groups, as shown in Table 2. The normal values for comparison have been given in Supplementary file 2.

**Table 2:** Comparison of the means of various electrocardiographic ventricular repolarization parameters and HRV parameters across the study groups.

Parameter	Hypothyroid (mean *±* SD)	Normal (mean *±* SD)	Difference in Means (Δ)	*p* value
Tpe interval in ms	58.07 ± 9.21	53.80 ± 8.89	+ 4.27	0.039*
QT interval in ms	330.16 ± 25.30	334.97 ± 23.54	−4.81	0.568
QTc interval in ms	379.10 ± 25.82	380.45 ± 27.03	−1.35	0.946
Tpe/QT	0.18 ± 0.03	0.16 ± 0.03	+ 0.02	0.227
Tpe/QTc	0.15 ± 0.02	0.14 ± 0.03	+ 0.01	0.217
PR int in ms	152.03 ± 48.57	144.05 ± 25.16	+ 7.98	0.413
JT int in ms	231.60 ± 26.00	237.22 ± 23.42	−5.62	0.367
Avg RR in ms	781.13 ± 120.37	794.81 ± 121.82	−13.68	0.653
Med RR in ms	779.47 ± 122.31	792.58 ± 124.30	−13.11	0.672
SDRR in ms	46.61 ± 20.54	50.67 ± 14.80	−4.06	0.368
SDSD in ms	43.93 ± 29.14	49.80 ± 26.78	−5.87	0.262
RMSSD in ms	44.40 ± 29.27	49.36 ± 26.01	−4.96	0.324
pRR50 in %	23.11 ± 22.56	28.16 ± 22.81	−5.05	0.224
TOTAL POWER in ms^2^	2696.53 ± 2160.52	2906.98 ± 1698.80	−210.45	0.347
VLF POWER in ms^2^	791.75 ± 531.02	810.39 ± 722.78	−18.64	0.444
LF POWER in ms^2^	1015.10 ± 1601.32	784.08 ± 612.71	+ 231.02	0.788
HFPOWER in ms^2^	1650.42 ± 2336.03	1257.44 ± 993.42	+ 392.98	0.347
LF/HF ratio	1.38 ± 1.87	1.16 ± 1.39	+ 0.22	0.757

p value* significant at < 0.05. *Tpe T* peak to T end, *QTc* corrected QT interval, *VLF* very low frequency, *LF* low frequency, *HF* high frequency, *LF(nu)* low frequency in normalized units, *HF(nu)* high frequency in normalized units, *SD* standard deviation, *AVRR* average RR interval, *MEDRR* median RR interval, *SDRR* standard deviation of RR intervals, *SDSD* standard deviation of differences between adjacent RR intervals, *RMSSD* square root of the mean squared differences of successive RR intervals, *Prr50* percentage of adjacent RRs differing by more than 50 ms.

### Pearson linear correlation of HRV and electrocardiographic ventricular repolarization parameters with thyroid hormones and TSH

**Fig. 1:**
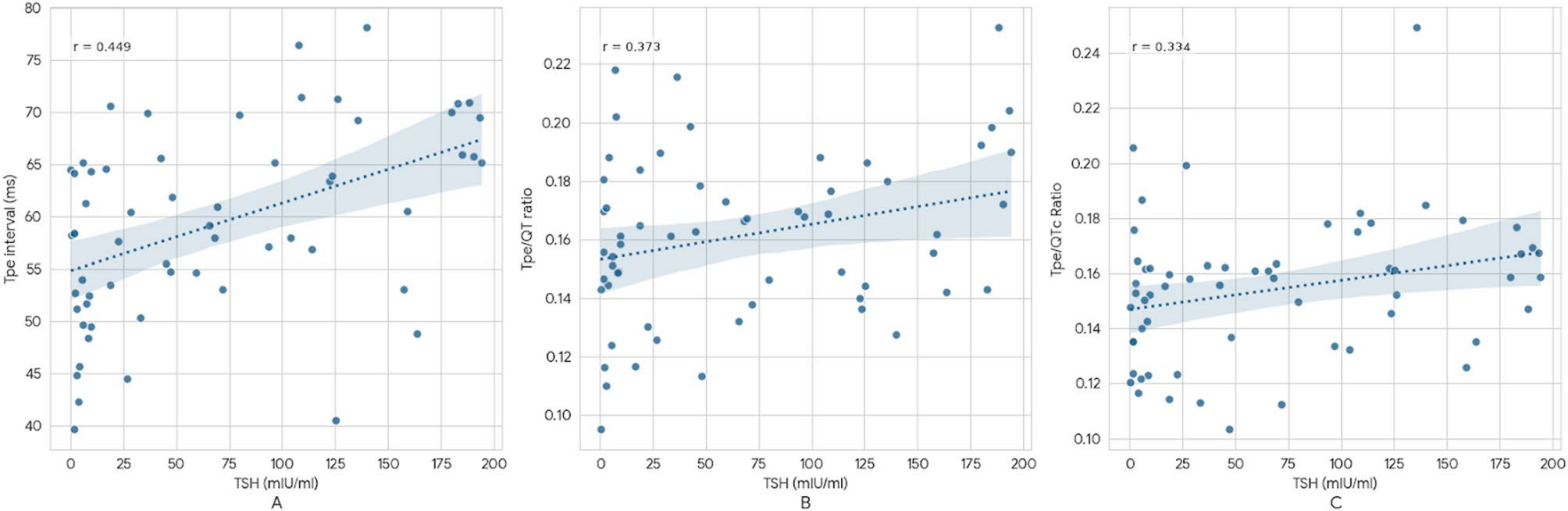
Graph showing the correlation between TSH and the Tpe interval (**A**). The Tpe/QT interval was positively correlated with TSH (**B**), and the Tpe/QTc interval was also positively correlated with TSH (**C**).

TSH was positively correlated with the Tpe interval, Tpe/QT ratio and Tpe/QTc ratio. No other correlation was detected between T4 and the parameters measured. The LF power percentage was correlated with the T3 hormone but not with the other parameters. The findings are shown in Table 3; Fig. 1. A subgroup analysis between the male and female pediatric populations was also done however the results were non-significant for the same.

**Table 3:** Pearson linear correlations of T3, T4 and TSH levels with ECG parameters, time domain parameters and frequency domain parameters.

	T3		T4		TSH	
	*r*	*p* value	*r*	*p* value	*r*	*p* value
Heart rate	−0.157	0.214	−0.098	0.443	0.131	0.301
Tpe interval	−0.217	0.085	−0.188	0.137	0.449**	< 0.001
QT interval	0.042	0.74	−0.028	0.824	0.055	0.663
QTc interval	−0.167	0.187	−0.167	0.187	0.218	0.083
TPe/QT ratio	−0.202	0.11	−0.149	0.239	0.373**	0.002
TPe/Qtc ratio	−0.139	0.272	−0.107	0.4	0.334**	0.007
PR interval	0.023	0.859	−0.015	0.909	0.024	0.849
JT Interval	0.078	0.542	−0.007	0.959	0.021	0.871
AVRR	0.143	0.258	0.063	0.621	−0.018	0.89
MEDRR	0.144	0.255	0.063	0.622	−0.013	0.921
SDRR	−0.008	0.951	−0.031	0.808	−0.07	0.584
SDSD	0.003	0.978	−0.006	0.963	−0.044	0.727
RMSSD	−0.016	0.902	−0.02	0.874	−0.034	0.788
PRR50	−0.014	0.911	−0.003	0.982	−0.03	0.813
Total Power	−0.013	0.916	−0.037	0.77	−0.02	0.878
VLF Power (ms^2^)	−0.078	0.541	0.003	0.982	0.038	0.763
LF Power (ms2)	−0.058	0.65	−0.123	0.334	0.111	0.384
HF Power (ms2)	−0.159	0.21	−0.141	0.267	0.14	0.27
LF/HF ratio	−0.028	0.825	−0.015	0.904	−0.02	0.878

p value* significant at < 0.05. *r* correlation coefficient, *BMI* body mass index, *WHR* Wasit-to-hip ratio, *WC* waist circumference, *Tpe* T peak-to-T end, *JT* interval between J-point and end of T-wave, *VLF* very low frequency, *LF* low frequency, *HF* high frequency, *LF(nu)* low frequency in normalized units, *HF(nu)* high frequency in normalized units, *SD* standard deviation, *AVRR* average RR interval, *MEDRR* median RR interval, *SDRR* standard deviation of RR intervals, *SDSD* standard deviation of differences between adjacent RR intervals, *RMSSD* square root of the mean squared differences of successive RR intervals, *Prr50* percentage of adjacent RRs differing by more than 50 ms.

The hypothyroid children were then followed up for ECG and HRV parameters after three months of thyroid therapy. We were able to follow up 23 out of 32 hypothyroid children after three months, as two were on antimalarial drugs and four were suffering from COVID-19 at the time of follow-up. Three patients decided not to follow up with their therapy. Therefore, we compared the pretreatment and post treatment parameters in 23 subjects, the results of which are shown in Tables 4 and 5. A post-hoc power analysis for the follow-up comparison (*n* = 23) showed that the study had 82% power to detect the observed difference in Tpe interval (effect size Cohen’s d = 0.73, α = 0.05).

**Table 4:** Anthropometric parameters, thyroid profile and blood pressure changes in 23 hypothyroid children before and after 3 months of thyroxine treatment.

Variable	Parameters at the time of diagnosis (mean *±* SD)	Parameters at 3 months Follow Up (mean *±* SD)	Difference in means (Δ)	*p* value
Weight in Kgs	40.13 ± 8.11	39 ± 7.99	−1.13	0.013*
Height in cm	131.13 ± 12.40	132.17 ± 12.19	+ 1.04	< 0.0001*
BMI in kg/m^2^	23.19 ± 2.62	22.16 ± 2.45	−1.03	0.001*
T3 (ng/ml)	0.48 ± 0.33	1.31 ± 0.42	+ 0.83	< 0.0001*
T4 (µg%)	2.60 ± 0.93	7.24 ± 1.49	+ 4.64	< 0.0001*
TSH (mIU/ml)	112.8 ± 49.26	5.26 ± 0.70	−107.54	< 0.0001*
SBP in mm Hg	108.69 ± 6.29	109.82 ± 4.37	+ 1.13	0.495
DBP in mm Hg	77.95 ± 4.81	77.82 ± 2.51	−0.13	0.898

p value* significant at < 0.05. *BMI* body mass index, *TSH* thyroid stimulating hormone, *SBP* systolic blood pressure, *DBP* diastolic blood pressure.

**Table 5:** Electrocardiographic parameters and heart rate variability parameters in 23 hypothyroid children before and after 3 months of thyroxine treatment.

Variable	Parameters at the time of diagnosis (mean *±* SD)	Parameters at 3 months Follow Up (mean *±* SD)	Difference in means (Δ)	*p* value
Heart Rate in bpm	80.38 ± 12.02	82.33 ± 10.46	+ 1.95	0.57
Tpe interval in ms	56.65 ± 9.92	50.56 ± 6.47	−6.09	0.001*
QT interval in ms	334.13 ± 20.35	325.58 ± 25.75	−8.55	0.250
QTc interval in ms	383.96 ± 18.24	377.80 ± 27.01	−6.16	0.35
Tpe/QT ratio	0.17 ± 0.03	0.15 ± 0.01	−0.02	0.006*
Tpe/QTc Ratio	0.14 ± 0.02	0.13 ± 0.01	−0.01	0.002**
PR interval in ms	136.42 ± 21.80	126.2 ± 15.99	−10.22	0.076
JT interval in ms	235.83 ± 19.03	228.58 ± 25.54	−7.25	0.31
Avg RR in ms	788.10 ± 129.08	791.12 ± 105.49	+ 3.02	0.92
Med RR in ms	785.13 ± 131.69	788.36 ± 104.23	+ 3.23	0.92
SDRR in ms	47.69 ± 22.91	41.91 ± 15.2	−5.78	0.38
SDSD in ms	46.83 ± 32.51	38.39 ± 19.16	−8.44	0.36
RMSSD in ms	47.46 ± 32.63	36.65 ± 18.08	−10.81	0.23
pRR50 in %	24.88 ± 24.07	12.62 ± 12.59	−12.26	0.05
TOTAL POWER in ms^2^	2662.37 ± 2230.30	3116.77 ± 1789.94	+ 454.40	0.54
VLF POWER in ms^2^	834.20 ± 571.90	970.08 ± 1825	+ 135.88	0.73
LF POWER in ms^2^	1176.99 ± 1858.16	1246.23 ± 1134.41	+ 69.24	0.86
HFPOWER in ms^2^	1906.75 ± 2589.82	1476.84 ± 1202.04	−429.91	0.52
LF/HF RATIO	1.12 ± 1.03	1.13 ± 1.06	+ 0.01	0.96

p value* significant at < 0.05. *Tpe* T peak to T end, *JT* interval between the J-point and end of the T-wave, *VLF* very low frequency, *LF* low frequency, *HF* high frequency, *LF(nu)* low frequency in normalized units, *HF(nu)* high frequency in normalized units, *SD* standard deviation, *AVRR* average RR interval, *MEDRR* median RR interval, *SDRR* standard deviation of RR intervals, *SDSD* standard deviation of differences between adjacent RR intervals, *RMSSD* square root of the mean squared differences of successive RR intervals, *Prr50* percentage of adjacent RRs differing by more than 50 ms.

## Discussion

In this study of 64 children aged 5–12 years, baseline anthropometric and cardiovascular parameters were comparable between hypothyroid and healthy groups, while thyroid profiles showed the expected severe biochemical hypothyroidism in the affected children. Most ECG and HRV indices did not differ significantly between groups, except for a modest prolongation of the Tpe interval in untreated hypothyroid children, suggesting associated changes in ventricular repolarization even in the absence of overt autonomic dysfunction. HRV time- and frequency-domain parameters were largely similar, indicating preserved autonomic modulation at diagnosis. Correlation analyses revealed that higher TSH levels were positively associated with Tpe interval, Tpe/QT, and Tpe/QTc ratios, highlighting a graded association pattern between the severity of hypothyroidism and repolarization parameters, while T3 showed a weak association only with LF%. After three months of thyroxine therapy in the 23 children out of the 32 children who completed follow-up, thyroid hormone levels normalized and significant improvements were observed in Tpe interval and related ratios, suggesting improvement in repolarization parameters following treatment. However, HRV parameters remained largely unchanged post-therapy, reinforcing the finding that overt autonomic alteration may not be prominent in pediatric hypothyroidism within this timeframe. The findings of this study align with those of prior studies, revealing a complex interplay between the thyroid gland and the heart, which has profound implications for both clinical diagnosis and treatment^4,17–21^.

### Hypothyroidism and autonomic dysfunction

Disruptions in thyroid function can have significant effects on cardiac physiology^22^. A previous study in adults highlighted the association between TSH levels and HRV, providing compelling evidence that thyroid dysfunction whether hyperthyroidism or hypothyroidism can impact cardiac autonomic regulation. However, the comparable HRV time- and frequency-domain parameters between groups indicate that autonomic modulation remains largely preserved at the point of diagnosis, and measurable dysfunction may develop only with longer disease duration or more severe hormonal deficiency^23^. Although previous studies have shown that HRV is a sensitive marker of thyroid dysfunction^24–28^, our study did not find any correlation between HRV parameters and thyroid status in the pediatric population which may be due to preserved autonomic modulation at the time of diagnosis. This might have occurred due to diagnosis at an early age or due to less severity of hypothyroidism in the population^4^. In hypothyroidism, metabolic rate and sympathetic drive are generally reduced, leading to alterations in autonomic balance^25,29,30^. However, despite these established physiological mechanisms, HRV parameters in the present study remained largely comparable between groups, suggesting preserved autonomic modulation at diagnosis in this pediatric cohort. Thus, alterations in TSH and thyroid hormone levels have the potential to influence autonomic control of the heart, influencing HRV and ultimately affecting overall cardiovascular function^22^.

### Hypothyroidism and ventricular repolarization

The Tpe interval is a crucial marker of ventricular repolarization, especially the transmural dispersion of ventricular repolarization^31^. In patients with thyroid dysfunction, particularly those with hypothyroidism, the Tpe interval is often prolonged, which has been reported as an electrophysiological association, as shown in a previous study^17^. As shown by previous studies by Alonso et al.^32^ and Fernandez et al.^33^, TSH has a direct effect on potassium ion channels, especially in the middle myocardial layers of the heart, affecting the transmural dispersion of ventricular repolarization. Our study findings shows that the reduced thyroid function leads to an extended Tpe interval, further highlighting the importance of thyroid regulation in cardiac electrophysiology. Furthermore, the authors reported that the Tpe interval after levothyroxine therapy in pediatric hypothyroid patients normalized to lower levels after 3 months of follow-up. These findings suggest a role for thyroid hormones and TSH in cardiac electrophysiology. These alterations in ECG readings may reflect underlying changes in cardiac electrical activity associated with thyroid function.

### Interplay of the variables

**Fig. 2:**
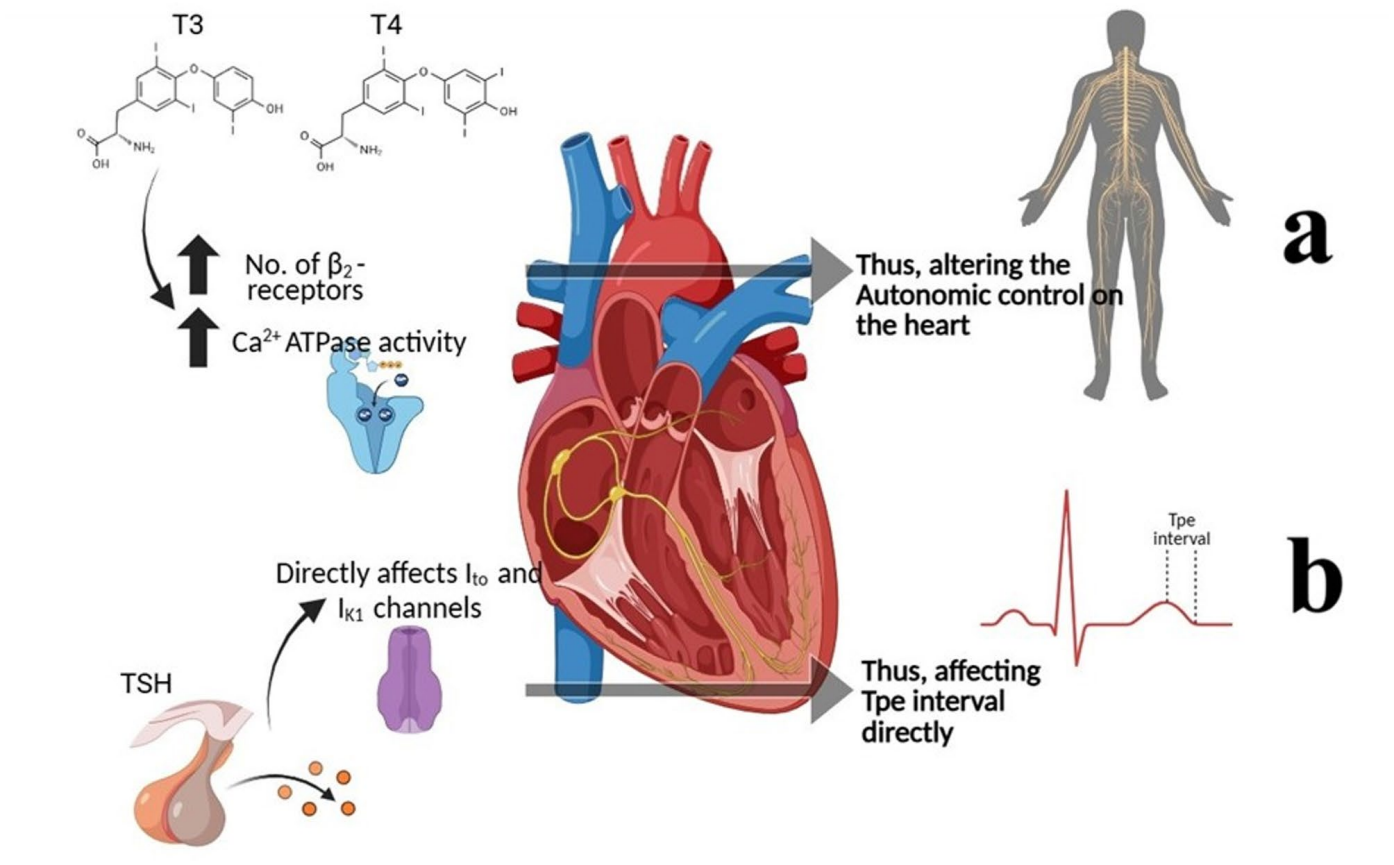
Effects of T3 and T4 on the heart and their effects on the autonomic nervous system and thus on HRV parameters (**a**) and the effects of TSH on specific K^+^ channels present in the mid-myocardial layer of the heart and thus affect the Tpe interval (**b**). (Image created in Microsoft PowerPoint).

The connections among thyroid hormones, HRV and ECG findings underscore the importance of the autonomic nervous system in regulating cardiac function in hypothyroidism. The influence of the thyroid gland on the cardiovascular system is also mediated through its impact on autonomic tone^34^. Both the sympathetic and parasympathetic branches of the autonomic nervous system are involved in the modulation of heart rate and cardiac output^35^. Some previous studies highlights how autonomic imbalances have been associated with cardiovascular electrophysiological alterations in previous studies^6,31^. Given the role of thyroid hormones in modulating autonomic balance, clinicians must consider thyroid dysfunction when evaluating patients with abnormal HRV or ECG findings, and vice-versa. This study supports consideration of thyroid status when interpreting ECG or HRV findings in pediatric populations. It highlights Tpe interval and HRV as noninvasive electrophysiological and autonomic markers, the clinical implications of which require further investigation. Monitoring these metrics guides treatment to restore thyroid levels and minimize cardiovascular risks, particularly in hypothyroid pediatric population. A figure showing the effect of TSH, T3 and T4 on the cardiac autonomic function has been shown in Fig. 2.

### Limitations

This study has several limitations that merit consideration. First, the relatively small sample size reduces the statistical power of the analysis and may limit the generalizability of the findings to the broader population. Second, participant dropouts during the follow-up period may introduce attrition bias, potentially influencing the robustness of the longitudinal data. Third, the analysis involved multiple parameters and correlations without formal correction for multiple testing, increasing the potential risk of type I error; therefore, the findings should be interpreted as hypothesis-generating. Finally, the clinical interpretation of the observed repolarization abnormalities is limited by the fact that actual arrhythmic events, such as PVCs or ectopy burden, were not assessed in this cohort.

## Conclusion

In conclusion, this study provides valuable evidence that thyroid dysfunction is associated with measurable changes in cardiac physiology, particularly through alterations in HRV and ECG parameters. These findings highlight the critical role of thyroid hormones in regulating autonomic balance and maintaining cardiovascular health. By demonstrating associations between TSH and ECG-derived parameters, while HRV measures remained largely preserved, we contribute to a growing body of evidence that underscores the need for a holistic approach to patient management, where thyroid function is considered a potential factor in cardiovascular pathology. Future research should continue to explore the underlying mechanisms of thyroid-cardiac interactions, with an emphasis on identifying effective strategies for improving outcomes in patients with thyroid-related cardiovascular conditions.

## Supplementary Information

Below is the link to the electronic supplementary material.

Supplementary Material 1

Supplementary Material 2

## Data Availability

The datasets used and/or analyzed during the current study are available from the corresponding author upon reasonable request.

## References

[1] Chaker, L., Bianco, A. C., Jonklaas, J. & Peeters, R. P. Hypothyroidism. *Lancet Lond. Engl.* **390**, 1550–1562. 10.1016/S0140-6736(17)30703-1 (2017). 10.1016/S0140-6736(17)30703-1 PMC6619426 28336049

[2] Taylor, P. N. et al. Global epidemiology of hyperthyroidism and hypothyroidism. *Nat. Rev. Endocrinol. Nat. Publishing Group.* **14**, 301–316. 10.1038/nrendo.2018.18 (2018). 10.1038/nrendo.2018.18 29569622

[3] Chaker, L. et al. Hypothyroidism. *Nat. Rev. Dis. Primer* **8**, 1–17. 10.1038/s41572-022-00357-7 (2022). 10.1038/s41572-022-00357-7 35589725

[4] Brusseau, V. et al. Heart rate variability in hypothyroid patients: A systematic review and meta-analysis. *PLoS ONE*. **17**, e0269277. 10.1371/journal.pone.0269277 (2022). 35657799 10.1371/journal.pone.0269277 PMC9165841

[5] Electrophysiology TF of the ES. Heart rate variability: standards of Measurement, physiological Interpretation, and clinical use. *Circulation* **93**, 1043–1065. 10.1161/01.CIR.93.5.1043 (1996). 8598068

[6] Stavrakis, S., Kulkarni, K., Singh, J. P., Katritsis, D. G. & Armoundas, A. A. Autonomic modulation of cardiac arrhythmias. *JACC Clin. Electrophysiol.* **6**, 467–483. 10.1016/j.jacep.2020.02.014 (2020). 32439031 10.1016/j.jacep.2020.02.014 PMC7370838

[7] Gupta, P. et al. Tp-e/QT ratio as an index of arrhythmogenesis. *J. Electrocardiol.* **41**, 567–574. 10.1016/j.jelectrocard.2008.07.016 (2008). 18790499 10.1016/j.jelectrocard.2008.07.016

[8] Antzelevitch, C. et al. Does Tpeak-Tend provide an index of transmural dispersion of repolarization? *Heart Rhythm Off J. Heart Rhythm Soc.* **4**, 1114–1119. 10.1016/j.hrthm.2007.05.028 (2007). 10.1016/j.hrthm.2007.05.028 PMC1994816 17675094

[9] Akın, A. et al. Left and right ventricular functions May be impaired in children diagnosed with subclinical hypothyroidism. *Sci. Rep.* **10**, 19711. 10.1038/s41598-020-76327-4 (2020). 33184320 10.1038/s41598-020-76327-4 PMC7661521

[10] Sawarthia, P., Bhosle, D. & Kalra, R. A prospective observational study to evaluate cardiovascular changes in patients of hypothyroidism. *Cureus* **15**, e40201. 10.7759/cureus.40201 (2023). 37435246 10.7759/cureus.40201 PMC10331040

[11] Ker, J. Thyroxine and cardiac electrophysiology—a forgotten physiological duo? *Thyroid Res.* **5**, 8. 10.1186/1756-6614-5-8 (2012). 22913316 10.1186/1756-6614-5-8 PMC3441891

[12] Tomar, A. et al. Analysis of ventricular repolarization parameters and heart rate variability in obesity: a comparative study. *Sci. Rep. Nat. Publishing Group.* **14**, 25855. 10.1038/s41598-024-76580-x (2024). 10.1038/s41598-024-76580-x PMC11519358 39468214

[13] Shaffer, F. & Ginsberg, J. P. An overview of heart rate variability metrics and norms. *Front. Public. Health*. **5**, 258. 10.3389/fpubh.2017.00258 (2017). 29034226 10.3389/fpubh.2017.00258 PMC5624990

[14] Li, Z-D. et al. Association between ventricular repolarization variables and cardiac diastolic function: A cross-sectional study of a healthy Chinese population. *World J. Clin. Cases*. **7**, 940–950. 10.12998/wjcc.v7.i8.940 (2019). 31119139 10.12998/wjcc.v7.i8.940 PMC6509266

[15] Waddell-Smith, K., Gow, R. M. & Skinner, J. R. How to measure a QT interval. *Med. J. Aust*. **207**, 107–110. 10.5694/mja16.00442 (2017). 28764626 10.5694/mja16.00442

[16] Kapoor, A. et al. Cardiovascular risks of hydroxychloroquine in treatment and prophylaxis of COVID-19 patients: A scientific statement from the Indian heart rhythm society. *Indian Pacing Electrophysiol. J.* **20**, 117–120. 10.1016/j.ipej.2020.04.003 (2020). 32278018 10.1016/j.ipej.2020.04.003 PMC7141642

[17] Aktar Ulukapi, N. et al. Evaluation of heart rate variability, QT dispersion, and Tp-e interval in pediatric subclinical hypothyroidism. *Pediatr. Res.*10.1038/s41390-024-03759-3 (2024). 39582062 10.1038/s41390-024-03759-3

[18] Gürdal, A. et al. Evaluation of Tp-e interval, Tp-e/QT ratio and Tp-e/QTc ratio in patients with subclinical hypothyroidism. *Ther. Adv. Endocrinol. Metab.* **8**, 25–32. 10.1177/2042018816684423 (2017). 28377800 10.1177/2042018816684423 PMC5363453

[19] Akın, A. et al. Evaluation of QT dispersion and Tp-e interval in children with subclinical hypothyroidism. *Pacing Clin. Electrophysiol. PACE*. **41**, 372–375. 10.1111/pace.13286 (2018). 29369370 10.1111/pace.13286

[20] Hoshi, R. A. et al. Linear and nonlinear analyses of heart rate variability following orthostatism in subclinical hypothyroidism. *Med. (Baltim).* **98**, e14140. 10.1097/MD.0000000000014140 (2019). 10.1097/MD.0000000000014140 PMC6358401 30681577

[21] Gupta, S. et al. Nerve conduction and heart rate variability in patients with hypothyroidism at a tertiary care centre in Eastern Nepal. *JNMA J. Nepal. Med. Assoc.* **56**, 407–411 (2017). 29453470

[22] Yamakawa, H. et al. Thyroid hormone plays an important role in cardiac function: from bench to bedside. *Front. Physiol.* **12**, 606931. 10.3389/fphys.2021.606931 (2021). 34733168 10.3389/fphys.2021.606931 PMC8558494

[23] de Miranda, É. J. F. P. et al. Relationship between heart rate variability and subclinical thyroid disorders of the Brazilian longitudinal study of adult health (ELSA-Brasil). *Braz J. Med. Biol. Res.* **51**, e7704. 10.1590/1414-431X20187704 (2018). 30156596 10.1590/1414-431X20187704 PMC6118047

[24] Kahaly, G. J. & Dillmann, W. H. Thyroid hormone action in the heart. *Endocr. Rev.* **26**, 704–728. 10.1210/er.2003-0033 (2005). 15632316 10.1210/er.2003-0033

[25] Bogdan, C. et al. Hypothyroidism and heart rate variability: implications for cardiac autonomic regulation. *Diagnostics* **14**, 1261. 10.3390/diagnostics14121261 (2024). 38928676 10.3390/diagnostics14121261 PMC11202468

[26] Syamsunder, A. N. et al. Decreased baroreflex sensitivity is linked to the atherogenic index, retrograde inflammation, and oxidative stress in subclinical hypothyroidism. *Endocr. Res.* **42**, 49–58. 10.1080/07435800.2016.1181648 (2017). 27260547 10.1080/07435800.2016.1181648

[27] Fagius, J., Westermark, K. & Karlsson, A. Baroreflex-governed sympathetic outflow to muscle vasculature is increased in hypothyroidism. *Clin. Endocrinol. (Oxf)*. **33**, 177–185. 10.1111/j.1365-2265.1990.tb00481.x (1990). 2225477 10.1111/j.1365-2265.1990.tb00481.x

[28] Syamsunder, A. N. et al. Association of sympathovagal imbalance with cardiovascular risks in overt hypothyroidism. *North. Am. J. Med. Sci.* **5**, 554–561. 10.4103/1947-2714.118921 (2013). 10.4103/1947-2714.118921 PMC3818829 24251274

[29] Mahajan, A. S., Lal, R., Dhanwal, D. K., Jain, A. K. & Chowdhury, V. Evaluation of autonomic functions in subclinical hypothyroid and hypothyroid patients. *Indian J. Endocrinol. Metab.* **17**, 460–464. 10.4103/2230-8210.111642 (2013). 23869303 10.4103/2230-8210.111642 PMC3712377

[30] Udovcic, M., Pena, R. H., Patham, B., Tabatabai, L. & Kansara, A. Hypothyroidism and the heart. *Methodist DeBakey Cardiovasc. J.* **13**, 55–59. 10.14797/mdcj-13-2-55 (2017). 28740582 10.14797/mdcj-13-2-55 PMC5512679

[31] Tomar, A. et al. The interplay of heart rate variability and ventricular repolarization parameters in the obese state: a review. *Cardiovasc. Endocrinol. Metab.* **14**, e00323. 10.1097/XCE.0000000000000323 (2025). 39802372 10.1097/XCE.0000000000000323 PMC11723674

[32] Alonso, H. et al. Thyroid stimulating hormone directly modulates cardiac electrical activity. *J. Mol. Cell. Cardiol.* **89**, 280–286. 10.1016/j.yjmcc.2015.10.019 (2015). 26497403 10.1016/j.yjmcc.2015.10.019

[33] Fernandez-Ruocco, J. et al. High Thyrotropin is critical for cardiac electrical remodeling and arrhythmia vulnerability in hypothyroidism. *Thyroid* **29**, 934–945. 10.1089/THY.2018.0709 (2019). 31084419 10.1089/thy.2018.0709 PMC6648210

[34] Olanrewaju, O. A. et al. Thyroid and its ripple effect: impact on cardiac structure, function, and outcomes. *Cureus* **16**, e51574. 10.7759/cureus.51574 (2024). 38318568 10.7759/cureus.51574 PMC10840038

[35] Gordan, R., Gwathmey, J. K. & Xie, L-H. Autonomic and endocrine control of cardiovascular function. *World J. Cardiol.* **7**, 204–214. 10.4330/wjc.v7.i4.204 (2015). 25914789 10.4330/wjc.v7.i4.204 PMC4404375

