# Supplementary material for: Evaluation and correlation of heart rate variability and ventricular repolarization parameters in an Indian pediatric clinical hypothyroid population: a prospective cohort study
Source: Sci Rep. 2026 Jan 29;16:6624. doi: 10.1038/s41598-026-36745-2 (PMC12913962; doi:10.1038/s41598-026-36745-2)
Supplement: Supplementary file 1 — Supplementary Material 1 [file 41598_2026_36745_MOESM1_ESM.docx]

**Supplementary file 1. Clinical presentation and implications of hypothyroidism**

|  | **Presentation** | **Signs and implications** |
| --- | --- | --- |
| **General metabolism** | Weight gain, cold intolerance, fatigue | Increase in body-mass index, low metabolic rate, myxedema*, hypothermia* |
| **Cardiovascular** | Fatigue on exertion, shortness of breath | Dyslipidaemia, bradycardia, hypertension, endothelial dysfunction or increased intima–media thickness*, diastolic dysfunction*, pericardial effusion*, hyperhomocysteinemia*, electrocardiogram changes* |
| **Neurosensory** | Hoarseness of voice, decreased taste, vision, or hearing | Neuropathy, cochlear dysfunction, decreased olfactory and gustatory sensitivity |
| **Neurological and psychiatric** | Impaired memory, paresthesia, mood impairment | Impaired cognitive function, delayed relaxation of tendon reflexes, depression*, dementia*, ataxia*, Carpal tunnel syndrome and other nerve entrapment syndromes*, myxedema coma* |
| **Gastrointestinal** | Constipation | [Reduced oesophageal motility, non-alcoholic fatty liver disease*, ascites (very rare)](https://www.sciencedirect.com/science/article/pii/S0140673617307031?via%3Dihub#tbl1fn1) |
| **Endocrinological** | Infertility and subfertility, menstrual disturbance, galactorrhoea | [Goiter, glucose metabolism dysregulation, infertility, sexual dysfunction, increased prolactin, pituitary hyperplasia*](https://www.sciencedirect.com/science/article/pii/S0140673617307031?via%3Dihub#tbl1fn1) |
| **Musculoskeletal** | Muscle weakness, muscle cramps, arthralgia | Creatine phosphokinase elevation, Hoffman's syndrome*, osteoporotic fracture* (most probably caused by overtreatment) |
| **Haemostasis and haematological** | Bleeding, fatigue | Mild anaemia, acquired von Willebrand disease*, decreased protein C and S*, increased red cell distribution width*, increased mean platelet volume* |
| **Skin and hair** | Dry skin, hair loss | Coarse skin, loss of lateral eyebrows*, yellow palms of the hand*, alopecia areata* |
| **Electrolytes and kidney function** | Deterioration of kidney function | [Decreased estimated glomerular filtration rate, hyponatraemia*](https://www.sciencedirect.com/science/article/pii/S0140673617307031?via%3Dihub#tbl1fn1) |

* Uncommon presentation
