# Supplementary material for: Evaluation and correlation of heart rate variability and ventricular repolarization parameters in an Indian pediatric clinical hypothyroid population: a prospective cohort study
Source: Sci Rep. 2026 Jan 29;16:6624. doi: 10.1038/s41598-026-36745-2 (PMC12913962; doi:10.1038/s41598-026-36745-2)
Supplement: Supplementary file 2 — Supplementary Material 2 [file 41598_2026_36745_MOESM2_ESM.docx]

**1. Normal 5-Minute HRV Metrics (Healthy Adults)**

This table provides reference ranges for short-term (5-minute) Heart Rate Variability (HRV) recordings in healthy adults. These values are primarily derived from the landmark **Task Force of the European Society of Cardiology (1996)** and the extensive systematic review by **Nunan et al. (2010)**, which refined these norms for modern clinical use.

| **Metric** | **Full Name** | **Normal Reference Range (Mean ± SD)** | **Description & Clinical Significance** |
| --- | --- | --- | --- |
| **Time Domain** |  |  |  |
| **SDNN** | Standard Deviation of NN intervals | **50 ± 16 ms** | Global measure of HRV. Reflects overall autonomic regulation (both sympathetic and parasympathetic). |
| **RMSSD** | Root Mean Square of Successive Differences | **42 ± 15 ms** | Primary marker of **parasympathetic (vagal) tone**. Less influenced by respiratory rates than frequency metrics. |
| **pNN50** | Percentage of NN intervals >50ms | **~9.9% ± 9%** (Highly variable) | correlated with RMSSD; reflects parasympathetic activity. |
| **Frequency Domain** |  |  |  |
| **LF Power** | Low Frequency Power (0.04–0.15 Hz) | **519 ± 291 ms²** *(Absolute)*    **52 ± 10 n.u.** *(Normalized)* | Reflects a mix of sympathetic and parasympathetic activity (often considered baroreflex activity). |
| **HF Power** | High Frequency Power (0.15–0.4 Hz) | **657 ± 777 ms²** *(Absolute)*    **40 ± 10 n.u.** *(Normalized)* | Pure marker of **parasympathetic (vagal) activity**. Highly respiratory-dependent (Respiratory Sinus Arrhythmia). |
| **LF/HF Ratio** | Low Frequency / High Frequency Ratio | **2.8 ± 2.6** | Index of **sympathovagal balance**. Higher values suggest sympathetic dominance; lower values suggest parasympathetic dominance. |

**Key Reference:**

- **Nunan D, et al.** "A quantitative systematic review of normal values for short-term heart rate variability in healthy adults." *Pacing and Clinical Electrophysiology*. 2010;33(11):1407-1417.
- **Task Force of the European Society of Cardiology.** "Heart rate variability: standards of measurement, physiological interpretation and clinical use." *Circulation*. 1996;93(5):1043-1065.

**Normal Metrics: Tp-e Interval & Ratios**

The Transmural Dispersion of Repolarization (TDR) is clinically assessed using the **T-peak to T-end (Tp-e)** interval. Prolongation of these metrics is a marker for malignant ventricular arrhythmias (like Torsades de Pointes).

**Note on Measurement:** These values are typically measured in precordial leads, ideally **V5** as these reflect the transmural axis best.

| **Metric** | **Normal Mean (Healthy Adults)** | **Upper Limit of Normal (95th Percentile)** | **Clinical Significance** |
| --- | --- | --- | --- |
| **Tp-e Interval** | **70 ± 12 ms** | **< 90 ms** | Time from the peak of the T-wave to the end of the T-wave (intersection of tangent slope). Represents dispersion of repolarization across the ventricular wall. |
| **Tp-e/QT Ratio** | **0.19 ± 0.03** | **< 0.25** | A ratio >0.25 is considered a highly sensitive predictor for ventricular arrhythmias (e.g., in Brugada, Long QT, or STEMI). |
| **Tp-e/QTc Ratio** | **0.17 ± 0.03** | **< 0.23** | Corrects for heart rate. Similar prognostic value to the uncorrected ratio; often used when heart rate varies significantly. |

**Key References:**

- **Koca B, et al.** "What is the Normal Value of Tpe Interval and Corrected Tpe Interval?" *Acta Medica*. 2020;51(4):10-15. (Defined the 90ms/100ms upper limits in healthy populations).
- **Castro Hevia J, et al.** "Tpeak-Tend and Tpeak-Tend dispersion as risk factors for ventricular tachycardia/ventricular fibrillation in patients with the Brugada syndrome." *J Am Coll Cardiol*. 2006;47(9):1828-1834. (Established the >0.25 ratio cutoff for risk).
